# Transitioning to sustainable, climate-resilient healthcare: insights from a health service staff survey in Australia

**DOI:** 10.1186/s12913-024-10882-8

**Published:** 2024-04-16

**Authors:** Andrea Huang, Susan Monro Cooke, Christine Garsden, Carol Behne, Erika Borkoles

**Affiliations:** 1 Griffith University, Brisbane, Australia; 2 Climate Action Beacon, Griffith University, Gold Coast, Australia; 3 Sunshine Coast Hospital and Health Service, Queensland Health, Sunshine Coast, Australia; 4 St Vincent’s Hospital, Melbourne, Australia; 5 Griffith Business School, Griffith University, Brisbane, Australia

**Keywords:** Climate change, Environmentally sustainable healthcare, Climate-resilient healthcare, Workforce, Survey, Health system transition, Healthcare culture, Health professions education and training

## Abstract

**Background:**

More than 80 countries, including Australia, have made commitments to deliver climate-resilient and low carbon healthcare. Understanding how healthcare workers view their own and their organization’s efforts to achieve sustainable and climate-resilient healthcare practice is vital to inform strategies to accelerate that transition.

**Methods:**

We conducted an online staff survey in a large state government hospital-and-health-service organisation in Queensland, Australia, to ascertain attitudes and practices towards environmentally sustainable, climate-resilient healthcare, and views about the organizational support necessary to achieve these goals in their workplace.

**Results:**

From 301 participants showed staff strongly support implementing sustainable and climate-resilient healthcare but require significantly more organizational support. Participants identified three categories of organizational support as necessary for the transition to environmentally sustainable and climate-resilient health services and systems: (1) practical support to make sustainability easier in the workplace (e.g. waste, energy, water, procurement, food, transport etc.); (2) training and education to equip them for 21st century planetary health challenges; and (3) embedding sustainability as ‘business as usual’ in healthcare culture and systems.

**Conclusions:**

The research provides new insight into health workforce views on how organizations should support them to realize climate and sustainability goals. This research has implications for those planning, managing, implementing, and educating for, the transition to environmentally sustainable and climate-resilient health services and systems in Queensland, Australia, and in similar health systems internationally.

**Supplementary Information:**

The online version contains supplementary material available at 10.1186/s12913-024-10882-8.

## Introduction

Growing planetary health challenges including climate change require that health systems rethink how to provide high quality, equitable health services, without further compromising human or environmental health [1–3]. Healthcare produces 4.4% of carbon emissions per year globally [4], and 7% in Australia [5], exacerbating climate change and harming health. Health system resilience in in the face of disruptive shocks such as pandemics and disasters, requires they continuously improve their abilities to adapt to extraordinary situations while maintaining delivery of care [6]. Climate-resilient, low carbon health systems can anticipate and adapt to climate-related shocks and stress, while minimizing greenhouse gas (GHG) emissions and other environmental harms to deliver quality care and protect health now and for future generations [7].

More than 80 countries, now including Australia, have made commitments to deliver climate-resilient and low carbon healthcare as part of the Alliance for Transformative Action on Climate and Health (ATACH) initiative [8]. The National Health Service (NHS) in England has been a leader, supported by a Sustainable Development Unit (SDU), now *GreenerNHS*, established to lead the NHS system transition following the UK Climate Change Act 2008 [9]. The NHS has committed to being carbon neutral by 2040 [10]. Australia established its National Health, Sustainability and Climate Unit in 2023 and launched its first National Health and Climate Strategy in December 2023 [11]. These welcome developments at federal government level will drive coordination across Australia’s seven states and territories and reinvigorate state and local (municipal) government-led policy and planning. For example, the state of Queensland’s Department of Health (QH) has committed to fostering a climate ready and environmentally sustainable health system and achieving zero net emissions by 2050 [12]. Queensland’s Human Health and Wellbeing Climate Change Adaptation Plan (H-CAP) was developed in 2018 [13] followed by Adaptation Planning Guidance for Hospitals and Health Services [14], Climate Risk Strategy 2021–2026 [12], and an Office of Hospital Sustainability in 2021.

The importance of healthcare staff in creating and maintaining an environmentally sustainable healthcare system is highlighted in the literature [15, 16]. Case studies demonstrate that small changes in workplace practice can result in significant reductions in waste and carbon emissions across a healthcare system, often resulting in financial savings that can be reinvested in healthcare [17–19]. Understanding staff attitudes, knowledge and practices across the health care system, including clinical and non-clinical roles, can expedite successful system transition [19, 20]. A survey by the Climate and Health Alliance (CAHA) of Australian health professionals’ views on climate change (*n* = 875), found most thought climate change was having a significant impact on public health (72%), and health services (57%), and that immediate action, and public education on climate change were needed (86%). While most (80%) agreed the health sector should lead the way on climate action and inform the public about the health effects of climate change, the study found key barriers to engaging in climate change action in the workplace were not feeling well enough informed about the health effects of climate change, and lack of support from their organization [21].

The purpose of this exploration of the attitudes, practices and support needs of staff in one of Queensland’s fifteen regional Hospital and Health Services (HHS) was to understand how healthcare workers in clinical and operational roles view their own and their organization’s efforts to achieve sustainable and climate-resilient healthcare, and to identify strategies to accelerate that transition.

## Materials and methods

### Design

A descriptive, cross sectional survey design with quantitative and qualitative aspects was chosen to ascertain (a) current staff attitudes, practices and organizational support, and (b) views on the support needed to implement environmentally sustainable, climate-resilient healthcare in their workplace. An anonymous, online survey was co-developed by research partners from the HHS, Griffith University and CAHA, based largely on three previous surveys [21–23]. It consisted of 25 closed ended items and 5 open ended questions providing opportunities for more nuanced responses. Items covered (1) Demographics; (2) Attitudes (a) sustainable healthcare and (b) climate-resilient healthcare; (3) Practices (at home; at work), (4) Organizational support (a) for sustainable practice, (b) climate-resilient behaviour and (c) what the organization could do to further support sustainable, climate-resilient practice. The survey was pilot tested for feasibility and content validity with twenty health service colleagues, and changes made to ensure it was easy to understand, of reasonable length (fifteen to twenty minutes), and that questions were neutral or unbiased. (See Appendix. *Survey: Attitudes, knowledge and practices of healthcare staff in Queensland Hospitals, regarding environmentally sustainable and climate-resilient healthcare)*.

### Study setting and participants

Situated in north-eastern Australia, Queensland is the second-largest, and third-most populous state, with a population of 5.3 million. Study participants were staff members at Sunshine Coast Hospital and Health Service (SCHHS), a large south-east Queensland HHS, serving a population approaching half a million. With approximately 10% of the state’s population Sunshine Coast is in one of the fastest growing regions in Queensland and encompasses major urban, inner and outer regional areas, with some pockets of significant socio-economic disadvantage and higher burden of disease. Full-time equivalent workforce is over 6000 [24]. Its 700-bed tertiary hospital has a Green Star Healthcare rating of 6 from the Green Building Council of Australia. The SCHHS joined the Global Green and Healthy Hospitals (GGHH) Pacific Network in 2020, and developed and launched its Environmental Sustainability Strategy 2021–2024, framed on the GGHH 10 goal agenda in 2021, the year preceding the survey. Its aim is to be ‘Australia’s cleanest greenest Hospital and Health Service’ by 2030, by ‘building a culture of waste avoidance, efficiency and innovation’ and ‘preparing for climate change’ [25]. SCHHS was recognised for leadership and climate resilience through the Health Care Climate Challenge 2021 [26].

### Data collection and analysis

Data collection was via LimeSurvey™. The goal was to achieve a broadly representative sample, of 362 respondents, based upon the reported staff population [24], a 95% confidence interval and an expected question response frequency of 50%. Supported by executive and department heads, the survey was promoted to staff by the Environmental Sustainability Committee members via staff email, newsletters, flyers, screensavers, noticeboards, a 30 s promotional video, and in person at staff forums, grand rounds, site visits and staff meetings. A prize draw of five digital gift cards was offered to encourage participation.

Responses were collated in a non-identifiable manner by LimeSurvey™. Data was exported to SPSS Statistics 28.0.1 for mainly descriptive analysis. Responses to open-ended questions were imported into NVivo20 for manual coding and analysis, to identify themes and add depth to the descriptive statistical analysis. Collected text responses were tested for word frequency to identify key concepts. Responses for each open-ended question were coded, and related codes grouped into themes [27].

### Ethics review

The study was conducted in accordance with the Declaration of Helsinki and approved by the Prince Charles Hospital Human Research Ethics Committee (TPCH HREC) (EC00168) Project ID 79,659, 18 Nov 2021, and Griffith University Human Research Ethics Committee GU Ref. No. 2022/003.

## Results

### Demographics and workplace information

After data cleaning, *n* = 301 complete responses with representation from all occupational groups and facilities were analysed. Table 1 summarises occupational and workplace information.

**Table 1:** Participant occupations and workplaces

Occupational group	Number	Percentage
Medical	17	5.6%
Nursing and Midwifery	119	39.6
Allied Health and Dental	57	18.9%
Administration and Building and Engineering Officers, Professional, Technical and Operational Officers	104	34.5%
Prefer not to say, Other	4	1.3%
**Total**	**301**	**100%**
**Facility**		
Tertiary Hospital	145	48.2%
Regional Hospitals (A, B, & C)	90	29.9
Community Health Services (x2) and Community Mental Health Unit	46	15.3%
Aged Care Facility	2	0.7%
Research and Education Institute	2	0.7%
Prefer not to say, Other (e.g., worked across multiple facilities)	16	5.4%
**Total**	**301**	**100%**

Most participants (74%) identified as female, 11% as male, and 15% chose not to identify. Average duration of employment in the health service was eight years. The largest age cohort (45%) were between 40 and 59 years, with 27% between 20 and 39 years, and 13% sixty years or over, with 15% undisclosed.

### Attitudes

Most participants (99.7%) had thought about the importance of the environment for people’s health and the majority ‘agreed’ or ‘strongly agreed’ that environmentally sustainable behaviour was important at home (92.5%), and at work (92.2%). Most (98.6%) had also thought about the interplay between climate change and health, the majority (72.9%) believing that climate change was already having a great or moderate impact on health in Queensland, and to a lesser extent on health services and infrastructure (60.4%), and their own practice or organization (49.4%). Most (77.8%) thought impacts on their services would increase a great or moderate amount, over the next ten years. Participants generally ‘agreed’ or ‘strongly agreed’ climate change is a serious problem requiring urgent action (90.4%) and that health services should take a leading role on climate change action (87%). The majority (91.9%) also ‘agreed’ or ‘strongly agreed’ that the public need to be better informed about the health impacts of climate change. Participants themselves reported varying levels of ‘feeling informed’ about climate change impacts on health with 24.4% feeling ‘not at all’, or ‘not very well informed’, 46% ‘somewhat informed’, and 29.4% ‘well’, or ‘very well informed’.

### Practices

**Fig. 1:**
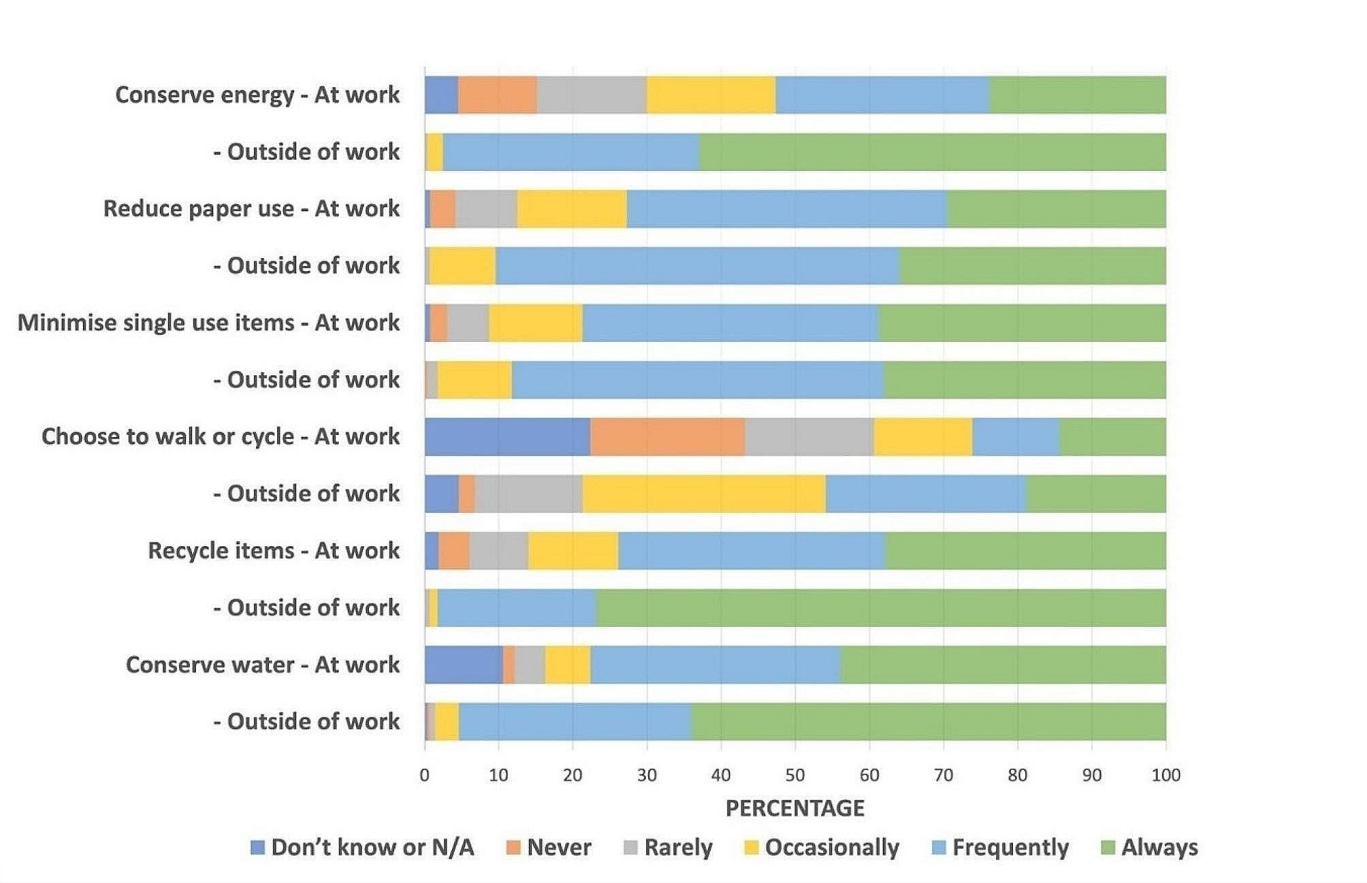
Practices for environmental sustainability at work and outside of workplace (n- 263–281)

From a given list of options, more sustainable behaviours were reported outside of work than in the workplace, particularly recycling, and conserving water and energy as demonstrated in Fig. 1.

The same pattern was reflected in communication behaviours related to climate change, with staff more likely to communicate more frequently (weekly or monthly) about climate change and health with friends and family (64%), than with colleagues (36%), leaders or managers in their organization (10%), or elected officials or community leaders (6%).

### Organizational values and support (perceived)

Three quarters of participants (74.5%) indicated it was important for the healthcare system and their organization to protect the environment, but over half (53.3%) felt they valued the environment more than their employer, and none felt their employer valued the environment more than they did. A Chi-Square Goodness of Fit test and post hoc analysis comparing this data to NHS SDU study data [23] showed a higher proportion of participants in this study, compared to the NHS participants, felt that they valued the environment more than their employer (*p* = 0.001). While nearly three quarters (68.8%) of participants indicated their employer encouraged them to protect the environment, over a quarter (27.7%) reported no organizational support. Four themes predominated in open ended responses. First, waste management particularly recycling, was mentioned by most:

Various recycling projects like metals, coffee cups, IV fluid bags. (Medical).

Second, a smaller proportion noted the new environmental policy and the work of the Environmental Sustainability Committee.

Environmental sustainability is now a strategic objective as part of our strategic plan…. and (we) have a very active environmental sustainability committee (Other, Finance).

Third, awareness of organizational support, and any change in organizational sustainability policies or practices in the past year, varied widely. For example,

It’s come a long way in the last 12 months (Nursing), contrasted starkly with:

None. Reached out to find out how to do more- limited commitment and limited enthusiasm (Other, non-clinical).

And lastly, environmental action was perceived to have been staff led rather than by the organization.

I believe there have been initiatives but these have been driven by individual staff members - not the organization (Other, Non-clinical).

Notably, general awareness of the HHS’s environmental policy was low. Only 19% were aware of the GGHH network and only 26.8% had seen the HHS Sustainability Strategy, launched mid 2021. In contrast, 61.8% were aware of ‘Choosing Wisely’ a longer standing campaign to reduce unnecessary tests, treatment and procedures, compatible with reducing costs, emissions and waste. There was little knowledge of climate risk assessment, preparation or organizational resilience-building. A majority (57.1%) did not know if climate action was taking place, and while 27.9% indicated that climate change action was happening or planned, only 2.1% thought their organization well-prepared for future impacts of climate change. Open ended comments supported quantitative results, for example:

I am not aware of any significant changes or plans that Queensland Health has made to address the challenges posed by climate change. (Allied Health).

### How could the organization support staff to make the healthcare system more environmentally sustainable?

**Fig. 2:**
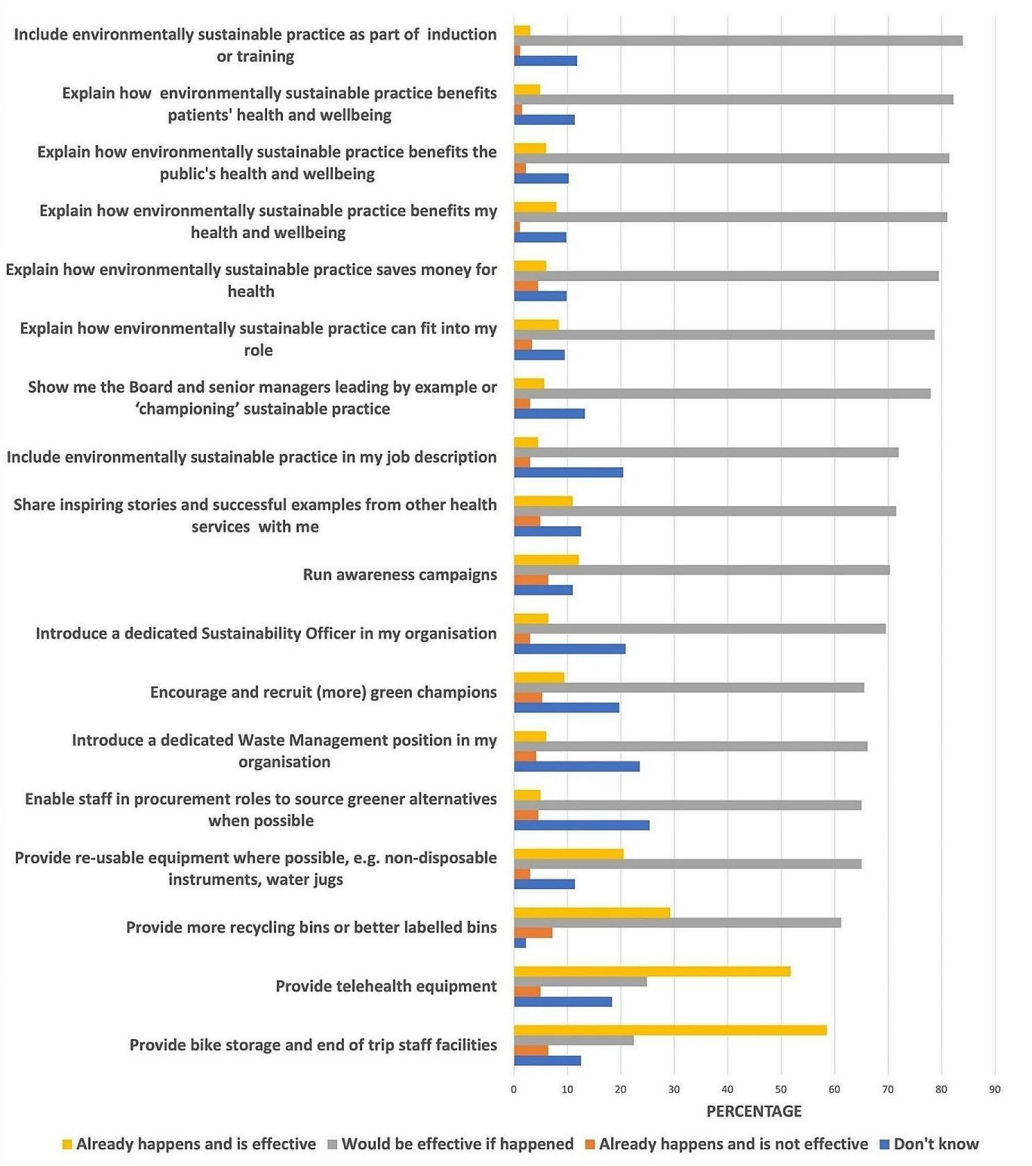
What can your organization do to support you to make the healthcare system more environmentally sustainable? Suggested strategies listed to left

Of eighteen strategies put forward, most respondents thought nearly all would be effective if implemented, suggesting many options for future action (see Fig. 2). Telehealth and end of trip facilities were seen as ‘happening’, and ‘effective’. Highly supported options (around 80%), related to staff education, training and information for example on benefits of sustainable practice for wellbeing and saving money for health. Actions to embed sustainability in operational structures and practices such as Sustainability roles, procurement, position descriptions, and management visibly championing sustainable practice were also popular (70 to 80%). More recycling bins and processes were desired by over 60%.

The open-ended follow-up question (‘Is there anything else that would support you to protect the environment at work?’) yielded reflections across three themes.

Reducing waste: The most frequently expressed need was for better recycling opportunities. Sustainable procurement, more reusable and low-carbon products were also desired.

Fundamentally the issue with hospital waste is the amount generated. People need to have access to appropriate recycling and be aware of what can and cannot be recycled (Medical).

A list of eco-friendly alternatives of supplies that we could order instead. (Allied Health).

Ditch Desflurane use in anaesthesia (Allied Health).

Secondly, education, training and information, including feedback from the organization on progress was highly valued:

Need education and support to adopt procedures and policies that reduce our environmental footprint at work (Allied Health).

Public signage; giving useful, simple stats against best practice e.g., carbon footprint compared to best practice. Also if we did reduce waste in some way, put that on the poster as well. (Medical).

The third theme encompassed transformative system and cultural change. Participants expressed the need to create a culture and system that values sustainability as highly as financial goals, with leadership, planning, staffing, resources and staff engagement at all health system levels.

Set up a whole-organizational culture of sustainability awareness relating to clinical and non-clinical resources and processes, through leadership, and involve all levels of staff. (Nursing).

The fact that minimising budget is considered far more important than sustainability practices…is the biggest problem (Nursing).

A dedicated Sustainability team, empowered to consult with departments and then facilitate the necessary changes, would be very helpful. (Other, clinical).

A gap analysis with an action plan and statewide agreed targets would be good. Even better if there was some funding to help implement change. (Medical).

Engage staff in auditing their local work area to identify and prioritise issues to promote sustainability…. (They) are the most suited to identifying issues which could have an impact. (Allied Health).

Follow the Green and Healthy Hospitals process, green ward competitions, brainstorm sessions to…give everyone a voice and stimulate innovation. (Nursing).

### Final comments

The final question elicited general reflections about the research project.

Would be good to hear the conclusions of your research, particularly if it helps influence environmental action (Medical).

Some articulated a sense of defeat about the scale and difficulty of sustainability challenges.

Unfortunately a lot of problems are past rectification…Infection management practices are extremely costly and cause much harm to the environment because of the single use of all consumables. (Other, non-clinical).

Others expressed hope and determination.

I hope that as an HHS we are able to have a more positive impact on our environment, for the sake of land, waterways and future generations. (Other, clinical, Aboriginal and Torres Strait Islander Health).

I really think addressing climate change needs to happen now…. otherwise future generations will bear the cost… We must listen to the scientists not politicians (Allied Health).

## Discussion

This research aimed to understand how clinical and operational healthcare workers in a large Southeast Queensland health service viewed their own and their organization’s efforts to achieve sustainable and climate-resilient healthcare, and to identify strategies to accelerate that transition.

### Pro-environmental staff attitudes, values and predispositions

The study showed most of the 301 participants in this Queensland cohort, who completed the thirty-item survey at the height of the COVID-19 pandemic, nevertheless had strong pro-environmental attitudes, values, personal behaviours, and enthusiasm to practice sustainable and climate-resilient healthcare. Over 90% were concerned that climate change was a serious problem needing immediate action, and that healthcare should lead on climate change action, with some open-ended responses calling for urgent climate change action. Climate change awareness was higher than previously reported in Australian health professions [21], adding to the growing evidence globally, of health professionals’ positive predisposition to implement low-carbon, sustainable, climate resilient healthcare to protect health [28]. However, consistent with previous Australian research, less than a third of our participants felt well informed about climate change impacts on health, reducing their capacity to educate patients, although most (over 91%) believe the community needs education to understand the connection between climate change and their health.

### Perceived disjunction between individual (staff) and organizational (HHS) values, attitudes and support for environmental sustainability in the workplace

Understanding barriers experienced by staff can inform solutions to accelerate transitions. The study showed participants practiced more sustainable behaviours outside the workplace than was possible at work, likely resulting in psychological discomfort, felt when one’s behaviour does not align with one’s values or beliefs [29]. Participants’ perception of lack of organizational commitment and support for sustainable healthcare practices is a barrier to successful system change, which requires alignment of staff and employer values and goals [30, 31]. In fact, a clear perception was that sustainability initiatives were led by staff rather than the organization. In a health workforce suffering from burnout and loss of meaning in their work [32], improving health professional experience is one of the quadruple aims of health services, alongside enhancing the patient experience, reducing costs, and improving healthcare outcomes [33].

The perceived lack of organizational support is partly explained by very low awareness of the HHS’s ambitious environmental and climate preparedness goals, Environmental Sustainability Strategy, and membership of GGHH, despite considerable communication efforts by the HHS Sustainability Committee. Awareness of any transformational change initiative takes time and attention to grow. Compared with the significantly higher awareness of the nine-year ‘Choosing Wisely’ campaign, low awareness of environmental goals and policy introduced only one year previously, and during COVID 19, is not surprising. Similarly, the even lower awareness of relatively new climate change adaptation policy and guidance.

### Empowering the transformation to sustainable and climate-resilient healthcare: pathways to success

Three pathways to success were distilled from analysis of responses about how the health organization could better support staff to reduce waste and carbon emissions and increase climate resilience.

#### Practical support to implement sustainable processes

Pandemic-related increases in disposable personal protective and testing equipment no doubt contributed to making waste, particularly recycling, the top sustainability issue for participants. As well as better physical resources (more recycling bins, waste streams, reusable equipment, better designed healthcare facilities) participants wanted useful information about reducing waste, tailored to their workplaces. Other sustainability domains, particularly energy, procurement, water, transport and food (waste) were also rated as priorities. While information is increasingly available through sustainable healthcare networks like GGHH and emerging communities of practice in Queensland and Australia, it is not yet consistent, uniformly applicable, or easily accessible in the workplace. The Australian Government’s first National Health and Climate Strategy now provides the policy authority and high-level directions to enable consistent reporting of health system emissions, and the development of a national health emissions reduction trajectory and decarbonisation roadmap [12]. National and jurisdictional climate and sustainable healthcare units must build on lessons from international and local experience, and work together to upscale systemic capacity, prevent reinvention, leverage economies of scale, and save time.

*Making it easy for staff to access practical, evidence-based information and processes to reduce emissions across routine sources such as energy, waste, water, transport, food, purchasing, buildings, chemicals and pharmaceuticals, will rapidly reduce emissions while reaping positive economic, social and health returns on investment.*

#### Education and training for 21st Century health challenges

Study participants wanted to learn how to ‘do better’ regarding sustainable healthcare. Providing ‘green’ (sustainability) training has been shown to increase commitment and voluntary engagement in pro-environmental behaviours in healthcare. Importantly, sustainability training was experienced by employees as organizational support, increasing their professional satisfaction [34]. Job satisfaction benefits employee wellbeing, workplace social sustainability and staff retention, as well as quality of care, health outcomes and patient satisfaction (Dawson, 2014 cited in [34] p.222). Healthcare providers must be equipped with the planetary health skills, values, knowledge and confidence to reduce healthcare emissions, prepare services to address climate related impacts, enable them to educate their patients and community, and advocate for action to meet Paris Climate Agreement commitments and the UN Sustainable Development Goals [35]. Sustainability and climate change education will also enhance eco-ethical leadership and governance for sustainability [36], and should be a requirement for all health roles, particularly executive and management [37]. Health impacts of climate change and principles of sustainable healthcare will be incorporated into Medical School and Teaching Hospital Standards in 2024 [38] and similar updates are happening in other health professions’ education. The National Health and Climate Strategy also encourages continuing professional development (CPD) for practicing health professionals [11]. Accessible, authoritative and affordable Australian CPD on these topics is currently limited [21].

*Addressing the need for training and education to build workforce capacity, particularly continuing professional development for existing practitioners should be a priority in Queensland as elsewhere.*

#### Embedding climate change and sustainability in healthcare culture and systems

A key insight from our research is the disjunction participants perceived between their own and their organization’s values, and its support for sustainability. Aboriginal and Torres Strait Islander Health participants succinctly articulated a planetary health aspiration for the HHS to have ‘a more positive environmental impact for the sake of land, waterways and future generations’. Participants generally articulated the need to embed sustainability and climate resilience as core business, much as ‘safety’ and ‘quality’ are core to healthcare and implicit in everyone’s roles. Leading Australian and international health practitioners and educators agree, arguing that sustainability must be adopted as a fundamental principle of quality healthcare, within a holistic planetary health paradigm, not an optional add-on ([39] p.9). Participants however perceived their organization did not value sustainability as highly as other goals, especially financial. Fortunately, evidence is growing that executive decisions which sustain momentum for improved organizational sustainability and climate resilience are beneficial on economic grounds as well as quality, service continuity, planetary health, and equity [1, 19, 40]. Jurisdictional and national health systems must therefore identify and secure the resources necessary to create and implement new climate-resilient and sustainable models of health care [41]. This includes leadership that engages and empowers staff as the UK’s experience in developing a sustainable healthcare system showed. Deliberate, effective staff engagement facilitates meaningful staff participation and is vital to creating practical solutions that protect health and build the mandate to implement them ([19] p.284). Many evidence-based programs exist, for example supporting ‘Net Zero’ leaders as system change agents to work with peer networks, and providing innovation grants can empower health professionals to develop scalable solutions that improve patient care and reduce healthcare’s environmental footprint [42, 43].

Strategies identified in our study can help organizations close the perceived gap between staff and organizational values, increase staff satisfaction, and create environments conducive to collaborative transformation. Australia’s Climate and Health Strategy sets out a whole-of-government plan of action for a sustainable, resilient, high quality, net-zero health system. It is underpinned by principles including One Health, Planetary Health, First Nations leadership, and Population Health and Prevention ([11] pp.6,7). It is vital that Australia’s governments, national and jurisdictional, now demonstrate genuine commitment to these values by resourcing and empowering the Strategy’s implementation. Without funding, little change in climate and health outcomes can be achieved.

*Health systems and services must embed sustainability as a core principle in workplace culture and practice, secure the resources necessary, and provide leadership that engages and empowers staff to participate in new sustainable models of care.*

### Strengths, limitations and future research

This cross-sectional study provides insight into staff attitudes, actions, perceptions and needs in one of Queensland’s health services leading the transition towards environmental sustainability and climate resilience. Greater participation by those more interested in environmental sustainability may have resulted in selection bias. Using items from previously validated instruments strengthened our survey’s validity, but its length at 15–20 min to complete, and its timing during the COVID-19 pandemic resulted in a lower sample size than desirable. More research is needed in smaller, geographically diverse, rural and remote locations which face additional challenges, and longitudinal research will further inform effective health system transition.

## Conclusion

The research provides new insight into health workforce views on how organizations should support them to realize climate and sustainability goals. In designing pathways forward, we can learn much from our own health workforce and the growing evidence and expertise in Australia and overseas. Organizational support strategies identified in this study will enhance staff satisfaction, increase engagement, and improve health, environmental, and financial outcomes, central goals for all health organizations. Nationally and jurisdictionally, more resourcing and facilitation are needed to provide locally relevant resources and encourage greater uptake. Investment is urgently warranted to reduce emissions and create sustainable, climate resilient healthcare at the scale and pace required to protect health now and for future generations.

### Electronic supplementary material

Below is the link to the electronic supplementary material.

Supplementary Material 1

## Data Availability

The data presented in this study are available on reasonable request from the corresponding author.

## Abbreviations

CAHA: Climate and Health Alliance
CPD: Continuing Professional Development
GGHH: Global Green and Healthy Hospitals
HHS: Hospital and Health Services
NHS: National Health Service (UK)
QH: Queensland Health Department
