# Supplementary material for: Transitioning to sustainable, climate-resilient healthcare: insights from a health service staff survey in Australia
Source: BMC Health Serv Res. 2024 Apr 16;24:475. doi: 10.1186/s12913-024-10882-8 (PMC11022411; doi:10.1186/s12913-024-10882-8)
Supplement: Supplementary file 1 — Supplementary Material 1 [file 12913_2024_10882_MOESM1_ESM.docx]

**Supplementary Materials: Survey Questionnaire**

**QUESTIONNAIRE**

**Staff Survey**

**Attitudes, knowledge and practices around environmentally sustainable and climate-resilient healthcare**

This research seeks to explore the attitudes, knowledge and practices of healthcare staff in [……….] hospitals, regarding environmentally sustainable and climate-resilient healthcare.

We want to know how we can support you to protect the environment, for example by using resources efficiently, reducing carbon emissions and reducing waste. We want to learn from you what actions you would like to take.

# About you and your organisation

We would like to know a bit more about you and the organization you work for so that we can compare the views of those working in different types of roles.

1. Which of the following occupational groups most closely matches your primary role? (Please select one answer)

Clinical

- 1. Medical
  2. Nursing
  3. Midwifery
  4. Dental
  5. Allied health
  6. Other (inc Laboratory Managers and Scientists) – please specify *free text box*

Non-clinical

- 1. Administration Officer
  2. Building and Engineering Officer
  3. Operational Officer
  4. Professional Officer
  5. Technical Officer
  6. Prefer not to say
  7. Other - Please specify *free text box*

1. What department do you work in?

*Free text box*

1. How many years/months have you worked for your current organisation?

*…. Years ………. Months*

1. In which of the following facilities are you mainly based? (Please select one answer)
   1. [List relevant health service facilities]
   2. [Facility]
   3. [Facility]
   4. [Facility]
   5. [Facility]
   6. etc
   7. Other - Please specify *free text box*
   8. Prefer not to say

# The environment and health

1. How much have you thought about the importance of the environment for people’s health?
   1. Not at all
   2. A little
   3. A moderate amount
   4. A great deal
2. To what extent do you agree that individual behaviour to protect the environment is important? (By protecting the environment we mean for example, using resources efficiently, reducing waste and reducing carbon emissions i.e. the release of greenhouse gases and/or their precursors into the atmosphere). (Please select one answer for each)

|  | Strongly agree | Agree to some extent | Disagree to some extent | Strongly disagree | Don't know |
| --- | --- | --- | --- | --- | --- |
| At work |  |  |  |  |  |
| Outside of work |  |  |  |  |  |

# Healthcare system

1. How important do you think it is for the healthcare system, and your organisation to protect the environment? (By healthcare system we mean […….] Health services and other providers of health care services including community and public health.) (Please select one answer)
   1. Very important
   2. Quite important
   3. Quite unimportant
   4. Very unimportant
   5. Don't know
2. Do your employer's environmental values (such as improving resource efficiency, reducing carbon emissions and reducing waste) fit with your own values? (Please select one answer).
   1. Yes, they are similar
   2. No, I value the environment more
   3. No, my employer values the environment more
   4. Don't know

1. Does your employer encourage you to do things that would protect the environment (such as recycling, energy saving, cycling, buying local or travelling by public transport)? (Please select one answer for each)
   1. Yes, definitely
   2. Yes, to some extent
   3. No
   4. Don’t know

1. Please provide examples of how your employer supports you to behave in environmentally sustainable ways.

*Free text box*

1. In the past year, has your organisation introduced any practices or policies aiming to improve its environmental sustainability (e.g. to improve resource efficiency, reduce carbon emissions, reduce waste)? Please specify.

*Free text box*

# Climate change and health

By ‘climate change’ we mean changes in the world’s climate that are due directly or indirectly to human activity and are in addition to natural climate cycles or variability.

1. How much have you thought about how climate change might affect people's health?
   1. Not at all
   2. A little
   3. A moderate amount
   4. A great deal
2. In your opinion, to what extent **is climate change already having an impact** upon:

|  | None at all | A little | A moderate amount | A great deal |
| --- | --- | --- | --- | --- |
| Queenslanders’ health |  |  |  |  |
| Health service infrastructure |  |  |  |  |
| The services in your own practice / organisation |  |  |  |  |

1. Over the **next 10 years**, to what extent do you think climate change will negatively impact upon:

|  | None at all | A little | A moderate amount | A great deal |
| --- | --- | --- | --- | --- |
| Queenslanders’ health |  |  |  |  |
| Health service infrastructure |  |  |  |  |
| The services in your own practice / organisation |  |  |  |  |

1. In your opinion, how prepared is your organisation for future impacts of climate change?
   1. Not prepared at all
   2. Somewhat prepared
   3. Well prepared
   4. Don’t know
2. To what extent do you agree with the following statements?

|  | Strongly disagree | Disagree | Neither agree or disagree | Agree | Strongly agree |
| --- | --- | --- | --- | --- | --- |
| Climate change is a serious problem that we need to take immediate action on |  |  |  |  |  |
| The public need to be better informed about the impacts of climate change on health |  |  |  |  |  |
| Health services and health organisations should help lead the way on climate change action |  |  |  |  |  |

1. How well informed do you feel about impacts of climate change on human health?
   1. Not at all informed
   2. Not very well informed
   3. Somewhat informed
   4. Well informed
   5. Very well informed
2. Taking action to tackle climate change is already happening or is planned for in your organisation.
3. Yes
4. No
5. Don't know

1. If you answered YES above, what is your organisation doing, or planning to do? Please indicate all that apply.

|  | Yes | No | Don’t know |
| --- | --- | --- | --- |
| Taking actions to reduce its own emissions |  |  |  |
| Providing training to staff on climate change |  |  |  |
| Assessing climate risk to Health Service Infrastructure |  |  |  |
| Assessing climate risk to our organisation's operational capacity |  |  |  |
| Assessing climate risk to our staff |  |  |  |
| Assessing climate risk to our community's health |  |  |  |
| Developing a HHS climate change adaptation plan |  |  |  |
| Other. Please specify other climate-readiness actions that your organisation is taking, not listed here. | *Free text box* | | |

# What do you do at home?

1. **Outside of work,** how frequently do you do the following? (Please select one answer for each)

|  | Always | Frequently | Occasionally | Rarely | Never | Don’t know N/A |
| --- | --- | --- | --- | --- | --- | --- |
| Conserve energy – e.g. Turn off lights and electronic equipment when I leave an empty room |  |  |  |  |  |  |
| Use renewable energy sources (e.g. solar, buy GreenPower) |  |  |  |  |  |  |
| Recycle items when appropriate rather than throw them away |  |  |  |  |  |  |
| Choose to walk or cycle instead of using a car when feasible |  |  |  |  |  |  |
| Minimise single use plastics and packaging e.g. use a ‘keep cup’, non-disposable cutlery, not accepting plastic bags or buying food with excess packaging |  |  |  |  |  |  |
| Reduce paper use by printing less, or keeping digital copies |  |  |  |  |  |  |
| Conserve water, for example turn off taps, fix leaks, use less water on showers, lawn etc. |  |  |  |  |  |  |
| Buy eco-friendly products and brands |  |  |  |  |  |  |
| Buy seasonal or locally grown food |  |  |  |  |  |  |

**What do you do at work?**

1. **At work,** how frequently do you do the following? (Please select one answer for each)

|  | Always | Frequently | Occasionally | Rarely | Never | Don’t know/NA |
| --- | --- | --- | --- | --- | --- | --- |
| Conserve energy – Turn off lights and electronic equipment when I leave an empty room |  |  |  |  |  |  |
| Recycle items when appropriate rather than throw them away |  |  |  |  |  |  |
| Choose to walk or cycle instead of driving to work when feasible |  |  |  |  |  |  |
| Reduce paper usage by printing less, keeping digital copies |  |  |  |  |  |  |
| Conserve water, for example turn off taps, fix leaks, use minimum amount required |  |  |  |  |  |  |
| Avoid travel by working from home if feasible, or using video or telephone conferencing |  |  |  |  |  |  |
| Minimise single use plastics and packaging e.g. use a ‘keep cup’, non-disposable cutlery, not accepting plastic bags or buying food with excess packaging |  |  |  |  |  |  |
| Advocate for procurement policy to source greener alternatives when purchasing supplies |  |  |  |  |  |  |
| Try to appropriately divide waste into clinical and non-clinical |  |  |  |  |  |  |

1. **What can your organisation do to support you** to make the healthcare system more environmentally sustainable? i.e. to help you improve resource efficiency, reduce carbon emissions and reduce waste (Please select one answer for each)

|  | Already happens and  is effective | Already happens and is not effective | Would be  effective if happened | Don't know |
| --- | --- | --- | --- | --- |
| Explain how environmentally sustainable practice saves money for health |  |  |  |  |
| Explain how environmentally sustainable practice can fit into my role |  |  |  |  |
| Include environmentally sustainable practice in my job description |  |  |  |  |
| Explain how  environmentally sustainable practice benefits patients' health and wellbeing |  |  |  |  |
| Run awareness campaigns |  |  |  |  |
| Explain how environmentally sustainable practice benefits the public's health and wellbeing |  |  |  |  |
| Explain how environmentally sustainable practice benefits my health and wellbeing |  |  |  |  |
| Include environmentally sustainable practice as part of  induction or training |  |  |  |  |
| Show me the Board and senior managers leading by example or ‘championing’ sustainable practice |  |  |  |  |
| Encourage and recruit (more) green champions |  |  |  |  |
| Share inspiring stories and successful examples from other health services with me |  |  |  |  |
| Introduce a dedicated Waste Management position in my organisation |  |  |  |  |
| Introduce a dedicated Sustainability Officer in my organisation |  |  |  |  |
| Provide re-usable equipment where possible, e.g. non-disposable instruments, water jugs |  |  |  |  |
| Provide more recycling bins or better labelled bins |  |  |  |  |
| Provide telehealth equipment |  |  |  |  |
| Provide bike storage and end of trip staff facilities |  |  |  |  |
| Enable staff in procurement roles to source greener alternatives when possible |  |  |  |  |

1. Is there anything else that would support you to act in a way that protects the environment at work?

*Free text box*

1. How often do you communicate (in-person, phone, email, letter, etc) about climate change and health with these groups or individuals?

|  | Never | Yearly | 2-3 times per year | Monthly | Weekly |
| --- | --- | --- | --- | --- | --- |
| Colleagues |  |  |  |  |  |
| Friends/family |  |  |  |  |  |
| Leaders/managers in your organisation |  |  |  |  |  |
| Elected officials or community leaders |  |  |  |  |  |

1. Is your hospital involved in the Global Green and Healthy Hospitals Network?
   1. Yes
   2. No
   3. Don’t know
2. Are you aware of the Choosing Wisely initiative to reduce unnecessary tests, treatments and procedures?
   1. Yes
   2. No
3. Have you seen the [……] Hospital and Health Service Sustainability Strategy 2021-2024?
   1. Yes
   2. No

# Background information

We would like to know a bit more about you so that we can compare the views of different types of staff.

1. Are you...?
   1. Male
   2. Female
   3. Prefer not to say
2. What is your age? (in years)

## Free text box

# Any other comments

1. And finally, are there any other comments you have about the study?

Thank you for completing the survey. Your views are much appreciated. Please now click 'Submit' button below to send us your response.

**Final screen – after pressing submit:**

**Thank you**

**Prize draw!**

Would you like to enter the draw to win 1 of 5 $100 gift cards? Please email us at: [emailaddress@gmail.etc]

**Interested in further involvement?**

Are you interested in future environmental sustainability initiatives at [Your Health Service]?

Please let us know! Email us at [yourhealthservicesustainability_link@etcetera]

to stay in the loop and become a sustainability champion in your department!
